# Are the outcomes of single-stage open reduction and Dega osteotomy the same when treating DDH in patients younger than 8 years old? A prospective cohort study

**DOI:** 10.1186/s10195-023-00725-3

**Published:** 2023-08-17

**Authors:** AboBakr Zein, Ahmed A. Khalifa, Mohamed Eslam Elsherif, Hassan Elbarbary, Mohamed Youness Badaway

**Affiliations:** 1 Orthopedics and Traumatology Department, Cairo University, Giza, Egypt; 2 Orthopaedic Department, Qena Faculty of Medicine, South Valley University, Kilo 6 Qena-Safaga Highway, Qena, 83523 Egypt; 3 Abo El-Reesh Hospital of Children, Cairo University, Cairo, Egypt

**Keywords:** Developmental dysplasia of the hip, DDH, Open reduction, Dega pelvic osteotomy, One-stage procedure

## Abstract

**Background:**

The primary objective was to report our early results after a one-stage procedure [open reduction (OR), Dega pelvic osteotomy (DPO), and femoral osteotomy (FO) when needed] for surgical management of a cohort of patients with developmental dysplasia of the hip (DDH). The secondary objective was to compare the functional, radiological, and complications among patients younger and older than 30 months.

**Materials and methods:**

This prospective cohort study included 71 hips with DDH in 61 patients with a mean age of 34.3 ± 19.5 months. All patients underwent one-stage surgical procedures, including OR + DPO and FO, if needed. Functional and radiographic assessment at the last follow-up was conducted using the modified Severin grading system and the Severin classification system, respectively, in addition to assessing the acetabular index (AI), osteotomies healing, and presence of complications. We divided patients into two groups, younger than 30 months (group I) and older than 30 months (group II).

**Results:**

We included 35 hips in group I and 36 in group II. All hips received OR + DPO, while 25 (69.4%) hips in group II had FO. The operative time was significantly longer in group II (103.19 ± 20.74 versus 72.43 ± 11.59 min, *p* < 0.001). After a mean follow up of 21.3 ± 2.3 months, the functional outcomes were satisfactory in 62 (87.3%) hips (94.3% in group I and 80.6% in group II, *p* = 0.35). There was a significant improvement in the AI in all patients compared with preoperative values (27.2° ± 2.9 versus 37° ± 4.2, *p* < 0.05). Furthermore, 63 (88.7%) hips had satisfactory radiographic outcomes (94.3% in group I and 83.3% in group II, *p* = 0.26), and all osteotomies showed radiographic healing. The overall complications incidence was significantly lower in group I compared with group II (5.7% versus 30.6%, *p* < 0.05), and avascular necrosis occurred in 4 (5.6%) hips, all in group II (*p* = 0.06).

**Conclusion:**

One-stage procedure entailing open reduction, Dega pelvic osteotomy, and femoral osteotomy when needed for managing DDH in patients younger than eight years old revealed acceptable clinical and radiological outcomes. However, there was a higher need for a concomitant femoral osteotomy in patients older than 2.5 years, and complications were more frequent.

*Level of evidence* III

## Introduction

Developmental dysplasia of the hip (DDH) involves a broad spectrum of abnormal hip development during infancy and early development, leading to a wide range of disease severity from mild acetabular dysplasia to frank hip dislocation [1–3]. Various management options were introduced for managing DDH, depending on the patient’s age and the severity of the disease; however, management after walking age poses a challenge to the treating surgeon owing to the development of adaptive changes related to the acetabulum, soft tissues, and possible increase in femoral anteversion [4, 5].

The main aim of DDH management is to obtain and maintain a concentric femoral head reduction within the acetabulum to permit normal hip development of the hip, which could be achieved by a wide range of non-operative and operative procedures with acceptable long-term outcomes; however, it is believed that the ease of treatment and the outcomes are inversely related to the age at presentation [1, 6–9].

Operative management entails various options, including open reduction (OR) and capsulorrhaphy, pelvic osteotomies (PO), and femoral osteotomies (FO) or combinations of all [1, 7, 10, 11]. However, most surgeons prefer to perform a one-stage procedure [open reduction, capsulorrhaphy, pelvic osteotomy, and a femoral osteotomy (shortening, derotation, or both) if needed] [5, 12–15], especially in children above 3 years old, as starting from this age the remodeling potential of the acetabulum becomes unpredictable [11, 16]. The one-stage procedure was reported to achieve acceptable functional and radiological outcomes in various studies, with the added benefit of saving the children from exposure to multiple surgical procedures, reducing hospital stays, and minimizing the socioeconomic burden [12, 13].

The primary objective of the current study was to report our early results after a one-stage procedure [open reduction, Dega pelvic osteotomy (DPO), and femoral osteotomy when needed] for surgical management of cohort of patients with DDH. The secondary objective was to compare the functional, radiological, and complications incidence among patients younger and older than 30 months.

## Materials and methods

A prospective cohort study was performed at Abo El-Reesh hospital of children (Cairo University, Egypt) after obtaining institue research board (IRB) approval (code no.: MD-215-2020) in the period between January 2019 to January 2020, where all patients presented with DDH (unilateral or bilateral) below eight years old were included. We excluded patients with previous hip surgical procedures, arthrogryposis, neuromuscular disorders (e.g., cerebral palsy), and septic hip sequelae.

Preoperative patients’ evaluation was performed through (1) history taking to predict if there was a positive family history and if previous management was attempted, (2) functional evaluation of the hip, gait, and leg length discrepancy (LLD), and (3) radiological evaluation after obtaining proper pelvis plain radiograph anteroposterior (AP) view. The degree of hip dislocation was assessed according to Tönnis classification [17] (Table 1), and the acetabular index (AI) was measured; we considered a normal value to be less than 28° in patients older than 6 months [18].

**Table 1:** Assessment criteria

Tönnis grades for DDH	
Grade I	Capital femoral epiphysis medial to Perkins line
Grade II	Capital femoral epiphysis lateral to Perkins line, but below the level of superior acetabular rim
Grade III	Capital femoral epiphysis at the level of the superior acetabular rim
Grade IV	Capital femoral epiphysis above the level of the superior acetabular rim
Functional assessment (modified Severin grading system)	
Grade I (satisfactory)	No pain, no limp, unlimited endurance
Grade II (satisfactory)	No pain, slight limp, slight restriction of endurance
Grade III (unsatisfactory)	Occasional pain, noticeable limp, endurance moderately restricted
Grade IV (unsatisfactory)	Regular pain, marked limp, severe restriction of endurance
Radiographic assessment (Severin classification system)	
Grade I (excellent) (satisfactory)	Normal hip CE angle > 15 degrees in children CE angle > 25 degrees in adults
Grade II (good) (satisfactory)	Mild deformity of head or neck Hip deeply and concentrically reduced CE angle as grade I
Grade III (fair) (unsatisfactory)	Dysplastic hips without subluxation CE angle < 15 degrees in children CE angle < 20 degrees in adults
Grade IV (poor) (unsatisfactory)	Subluxation
Grade V (poor) (unsatisfactory)	Head articulating with a false acetabulum in the upper part of the original acetabulum
Grade VI (poor) (unsatisfactory)	Redislocation
Kalamachi et al. femoral head AVN grades	
Grade I	Changes affecting the ossific nucleus
Grade II	Lateral physeal damage
Grade III	Central physeal damage
Grade IV	Total damage to the femoral head and physis

### Surgical details

Two senior surgeons (H.E. and A.B.Z.) performed all surgeries under general anesthesia while the patient was supine on a radiolucent table. After hip reevaluation under anesthesia and full draping, an adductor tenotomy was performed as a first step in all patients. The hip joint was approached through a curved anterolateral (Bikini) incision to perform an open reduction of the hip joint as a second step; this was achieved after clearing the capsule from the attached rectus muscle reflected head; furthermore, if the capsule superolateral part was adherent to the lateral wall of the ilium, it was bluntly dissected down to the superior edge of the acetabulum by a periosteal elevator or a Cobb dissector. Once the capsule became fully exposed, a T-shaped capsulotomy was performed through a transverse incision parallel to the true acetabulum margin and a vertical incision perpendicular to the first incision; the flaps were tagged by sutures. After clearing all soft tissue obstacles and identification of the true acetabulum, the femoral head was brought to the acetabulum by gentle traction, hip flexion, abduction, and internal rotation with pressure applied to the greater trochanter; if the hip was reduced and stable, only OR and capsulorrhaphy were performed by removing the redundant superolateral part of the capsule, then suturing the inferolateral to the medial part using running absorbable suture; however, if the hip was unstable or could not be reduced, the need for a PO or FO (which was performed through a separate lateral incision) or both were determined as follows:

1. DPO was added if the hip was unstable in flexion and abduction.
2. If the hip was unstable after performing internal rotation accompanying flexion and abduction, a derotational FO was added to DPO.
3. If the femoral head is reduced under tension with the leg in the neutral position, this indicates the need for a concomitant shortening FO.

For performing a femoral osteotomy, the femoral head was first reduced to the acetabulum by internal rotation and abduction and maintained in position with a temporary smooth K wire. A 4-hole 3.5 mm dynamic compression plate was placed on the proximal femur, and the proximal two screws were inserted, then a subtrochanteric femoral osteotomy was performed using an oscillating saw. The amount of derotation was determined according to the patella position; the distal femoral fragment was externally rotated till the patella was facing upward; if the hip reduction was under tension, a femoral shortening was performed, the amount of which was determined by the amount of overlap between the proximal and distal segments. Then the osteotomy was fixed by inserting the distal two screws.

After applying the above inclusion criteria, 82 hips were eligible for surgical intervention; 11 hips had only OR, and in 71 hips, DPO ± FO was performed as the surgical technique described in the literature [5, 19, 20]. In cases with bilateral hip affection, the surgery was performed sequentially, starting with the hip having a higher degree of dislocation; then, the other side was operated upon after 2–3 weeks intervals. Postoperatively, the patient was placed in a hip Spica for 12 weeks with the operated hip kept in 40° abduction, 60° flexion, and neutral rotation.

### Postoperative and follow up protocols

An immediate postoperative AP pelvis plain radiograph was performed to confirm the accuracy of hip reduction and acetabulum coverage. Usually, patients stayed in the hospital for 24 h, where the child’s mother or caregiver was given instructions about cast care.

The follow up visits were scheduled at 2 weeks for the wound check and the hygiene of casting. The second visit was at 6 weeks postoperatively for rechecking the cast and seeing if a change was needed. The third visit was after 3 months postoperatively for cast removal and radiographic assessment. Then the patient was sent for physiotherapy to start active mobilization, hydrotherapy, and gradual weight bearing. Afterward, the follow-up visits were scheduled at 6 and 12 months, then annually.

### Outcomes assessment (Table 1)

A functional assessment was performed according to the modified Severin grading system [21], and the LLD was measured as well. Radiographic assessment was performed according to the Severin classification system [22, 23], in addition to assessing the AI, Shenton line status, osteotomies healing, and presence of femoral head avascular necrosis (AVN), which was graded according to Kalamachi and MacEwen [24]. Complications at any time during the follow-up period were reported. All patients (71 hips) were available for assessment by the last follow-up, where the functional and radiological assessments were measured and reported. We divided patients into two groups, those younger than 30 months of age (group I) and patients older than 30 months of age (group II).

### Statistical analysis

The sample size was calculated using G power software version 3.1.3, using the paired *t*-test for comparing the difference of AI angle pre- and postoperatively with the following parameters: effect size 0.4 (Czubak et al. [5]), alpha error 0.05, power (1-beta error prob) 0.95, and one tailed. The minimum required sample size was 70 hips, which was increased to 75 to compensate for possible dropouts. Data was collected and analyzed using SPSS (Statistical Package for the Social Science, version 20, IBM, and Armonk, New York). Normal data distribution was determined using the Shapiro test. Quantitative data were expressed as mean ± standard deviation (SD) and compared by Student’s *t*-test or Mann–Whitney *U* test in case of two different means, while ANOVA was used in case of more than two different means. Nominal data were presented as number (*n*) and percentage (%) and compared by the chi-squared test or the Fisher’s exact test. The confidence level was kept at 95%; hence, the *p* value was considered significant if < 0.05.

## Results

**Fig. 1:**
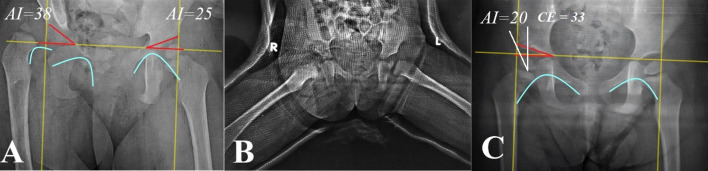
Female child, 18 months, presented with right DDH, Tönnis grade III. **A** preoperative. **B** Immediate postoperative. **C** After 20 months of follow-up, the radiographic result was Severin’s grade I

**Fig. 2:**
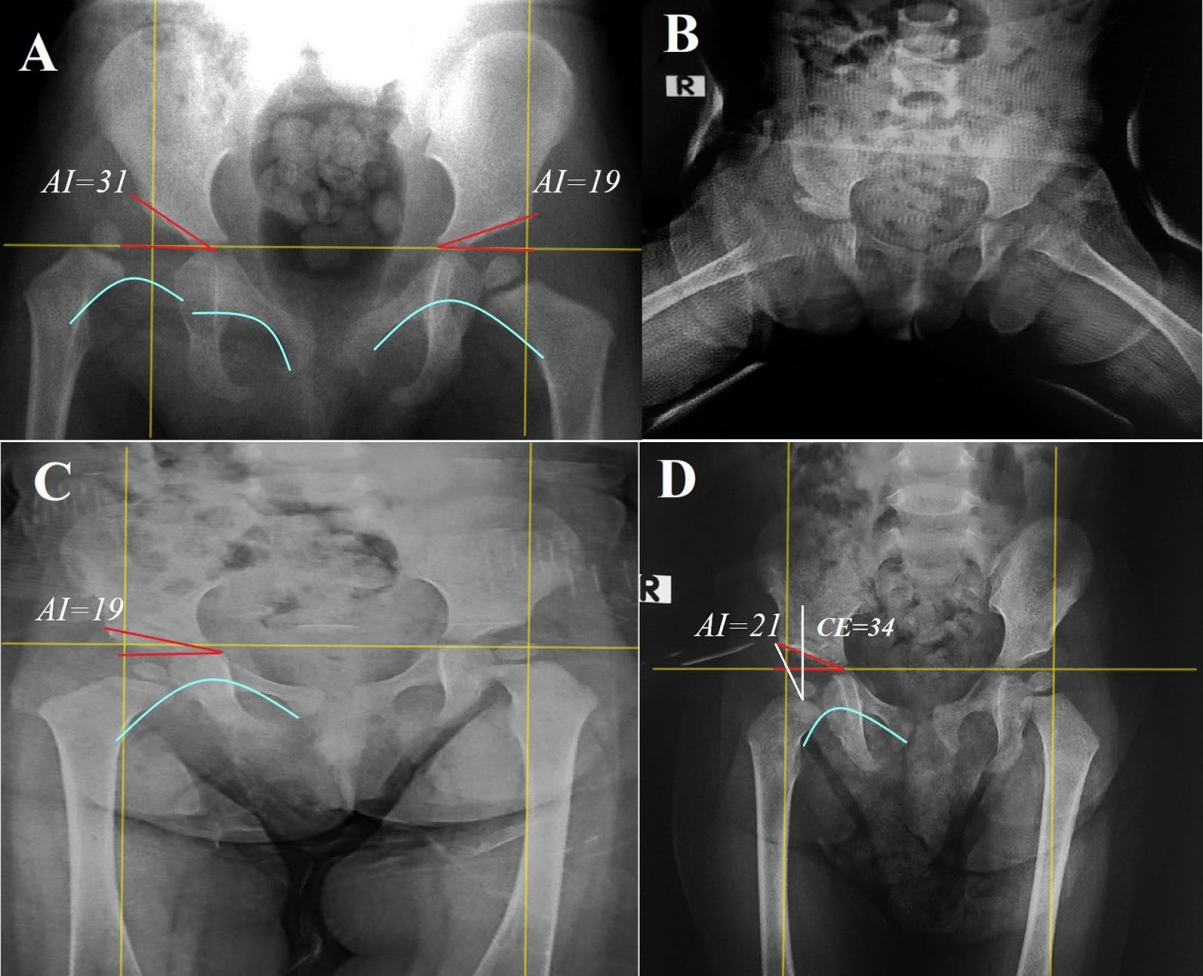
Female child, 20 months, presented with right DDH, Tönnis grade III. **A** preoperative. **B** Immediate postoperative. **C** After 6 months of follow-up, **D** after 18 months follow up, the radiographic result was Severin’s grade Ι

**Fig. 3:**
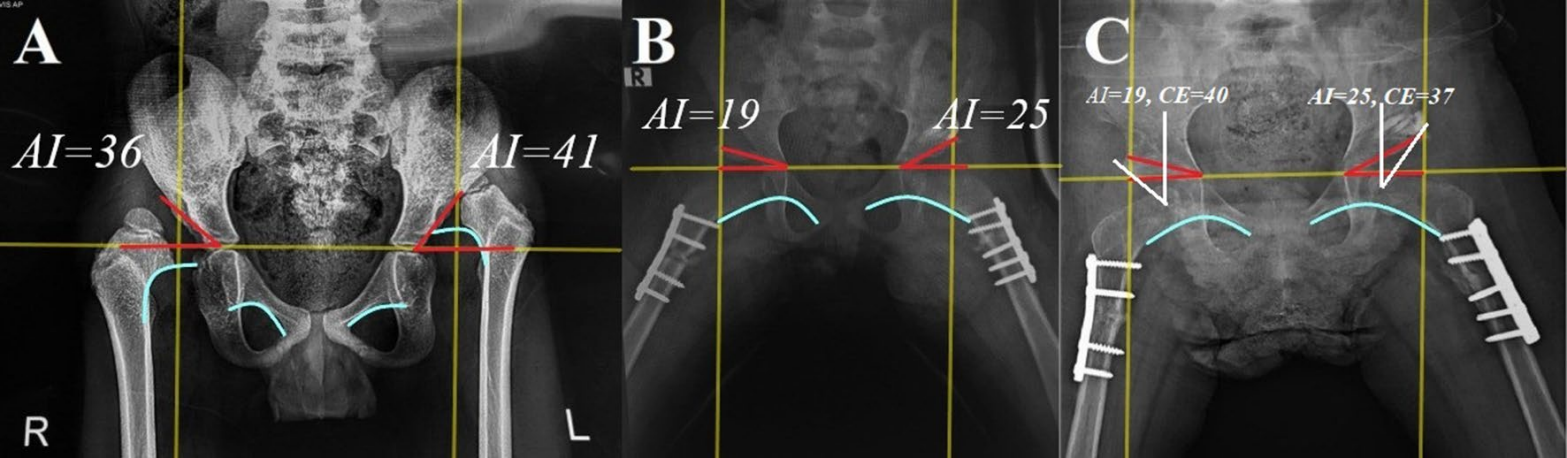
Male child, 72 months, presented with bilateral DDH, Tönnis grade III on the right side and grade IV on the left side. **A** preoperative. **B** Immediate postoperative. **C** After 18 months of follow-up the radiographic result was Severin’s grade I on the right side and grade II on the left side

**Fig. 4:**
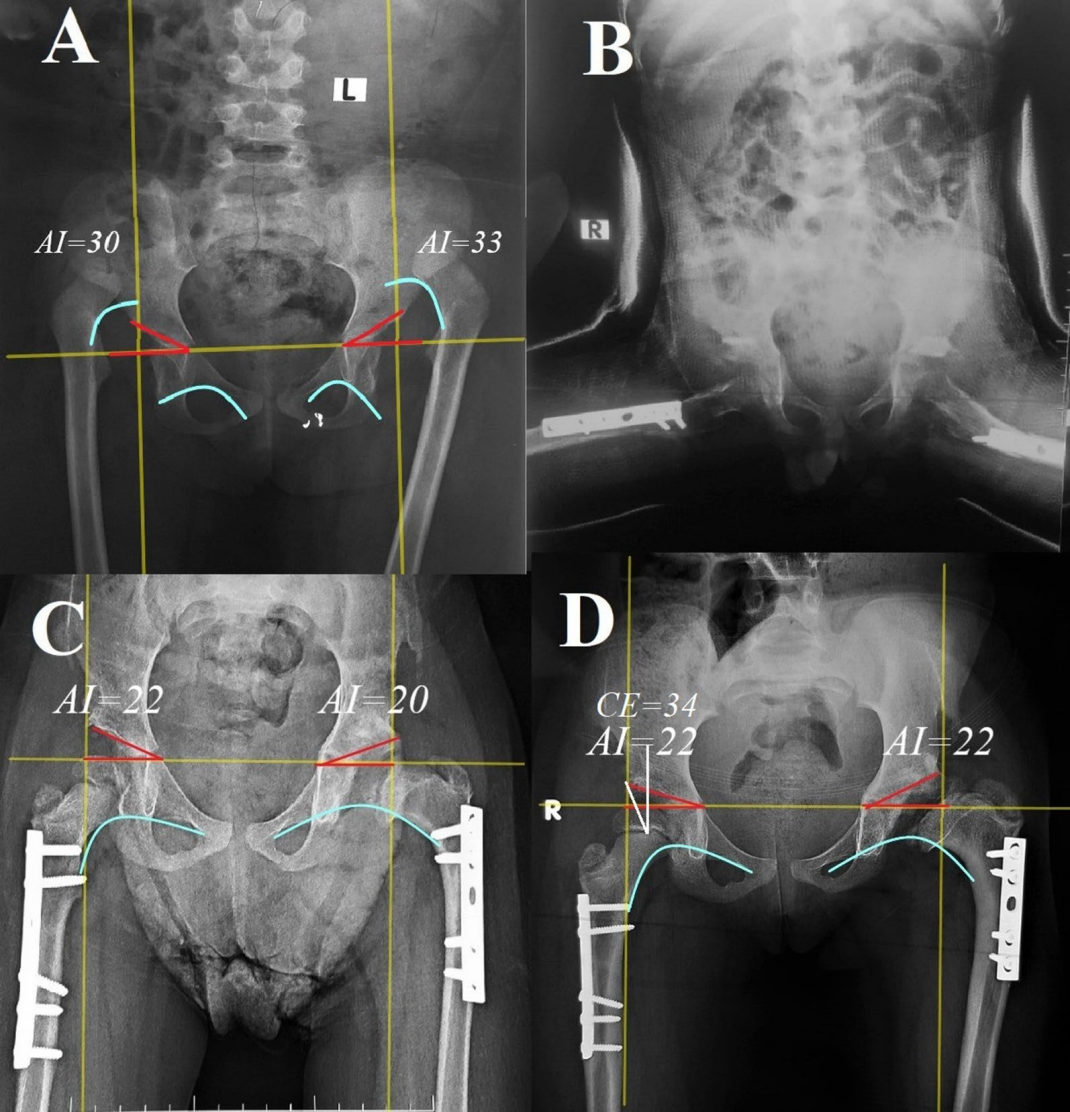
Female child, 70 months, presented with bilateral DDH, Tönnis grade IV. **A** preoperative. **B** Immediate postoperative. **C** After 6 months of follow-up. **D** After 18 months of follow-up. Full union of the osteotomies and the radiographic result was Severin’s grade I on the right side and grade III on the left side. The left hip shows Kalamchi and MacEwen AVN grade IV

Sixty-one patients (71 hips, 51 unilateral, and ten bilateral), with a mean age of 34.3 ± 19.5 months (12–84 months) at the time of surgery were included, 35 hips in group I and 36 in group II. A positive family history was found in four (5.6%) patients. All hips received a one-stage procedure (group I received OR + DPO, while 25 hips in group II had an additional FO) and were followed up for a mean of 21.3 ± 2.3 months (15–24 months). There was no difference regarding the basic demographic data except for age, with females and left-side affection representing the majority (Table 2). Regarding preoperative functional and radiographic assessment, there was a significant difference between both groups in the LLD and the Tönnis dislocation degrees (where both were higher in group II), with no difference regarding the AI (Table 3). Operative time was significantly longer in group II, where 25 (69.4%) hips underwent additional FO; however, the amount of blood loss was not different between both groups (Table 4). The functional outcomes measured during the last follow-up showed satisfactory outcomes in 62 (87.3%) hips according to modified Severin grade (94.3% in group I and 80.6% in group II), with no difference between groups. Although the mean postoperative LLD for all patients was significantly less in both groups compared with the preoperative values (1.6 ± 3.6 mm versus 5.5 ± 5.6 mm, *p* < 0.001), the postoperative LLD was less between patients in group I compared with group II (Table 5). Radiological outcomes at the last follow-up showed significant improvement in the AI in all hips compared with preoperative values (27.2° ± 2.9 versus 37° ± 4.2, *p* < 0.001); however, there was no difference in the final AI between both groups (Figs. 1, 2, 3, and 4). Furthermore, 63 (88.7%) hips had satisfactory radiographic outcomes according to Severin radiological grades (94.3% in group I and 83.3% in group II), with no difference between both groups (Table 5), and all osteotomies showed radiographic healing. The overall complications incidence was significantly lower in group I compared with group II (5.7% versus 30.6%, *p* < 0.001); AVN was reported in four (5.6%) hips (Fig. 4D), where the incidence was higher in group II compared with group I (11.1% versus0%, *p* = 0.06). Details of the individual complications are presented in (Table 5). Additionally, as all the hips that underwent FO were in group II, when comparing patients within the group according to having a FO or not, we found that the operative time and blood loss were significantly higher in patients who had FO, 87.27 ± 9.04 min (70–100 min) versus 110.2 ± 20.64 min (90–200 min) and 75.45 ± 16.35 ml (50 to 100 ml) versus 98.8 ± 20.27 ml (60 to 150) (*p* < 0.001), respectively. However, there was no difference regarding functional, radiological, and complications incidence outcomes (Table 6).

**Table 2:** Baseline demographic data of studied patients

Parameter	Group I (*n* = 35)	Group II (*n* = 36)	*p* value
Age (months)^a^	19.08 ± 5.05 (12–29)	49 ± 16.89 (31–84)	**< 0.001**
Sex^b^			0.41
Male	7 (20%)	9 (25%)	
Female	28 (80%)	27 (75%)	
Affected side^b^			0.53
Right	16 (45.7%)	15 (41.7%)	
Left	19 (54.3%)	21 (58.3%)	
Follow-up (months)^a^	21.68 ± 2.24 (16–24)	21.08 ± 2.43 (15–24)	0.28

The bold emphasis indicates statistical significance

*p* value was significant if < 0.05

^a^Data expressed as mean ± SD (range)

^b^Data expressed as frequency (percentage)

**Table 3:** Preoperative clinical and radiological data

	Group I (*n* = 35)	Group II (*n* = 36)	*p* value
LLD (mm)^a^	4 ± 4.82 (0–15)	6.94 ± 6.01 (0–20)	**0.02**
AI^a^	36.37 ± 3.15 (30.40–44.10)	37.65 ± 2.60 (32.10–44.10)	0.06
Tönnis grade^b^			**< 0.001**
Grade II	3 (8.6%)	0	
Grade III	17 (48.6%)	8 (22.2%)	
Grade IV	15 (42.9%)	28 (77.8%)	

The bold emphasis indicates statistical significance

*LLD* leg length discrepancy, *AI* acetabular index

*p* value was significant if < 0.05

^a^Data expressed as mean ± SD (range)

^b^Data expressed as frequency (percentage)

**Table 4:** Intraoperative data

	Group I (*n* = 35)	Group II (*n* = 36)	*p* value
Blood loss (ml)^a^	56.57 ± 19.08 (30–100)	47.67 ± 34.67 (10–90)	0.18
Operative time (min)^a^	72.43 ± 11.59 (50–100)	103.19 ± 20.74 (70–200)	**0.001**
Femoral osteotomy^b^	0	25 (69.4%)	**< 0.001**
Pelvic osteotomy^b^	35 (100%)	36 (100%)	–

The bold emphasis indicates statistical significance

*p* value was significant if < 0.05

^a^Data expressed as mean ± SD (range)

^b^Data expressed as frequency (percentage)

**Table 5:** Postoperative outcomes measured at the last follow-up

	Group I (*n* = 35)	Group II (*n* = 36)	*p* value
Clinical outcomes			
LLD (mm)^a^	1.17 ± 0.26 (0–5)	4.52 ± 3.05 (0–15)	**< 0.001**
Modified Severin grade^b^			0.35
Grade I	23 (65.7%)	20 (55.6%)	
Grade II	10 (28.6%)	9 (25%)	
Grade III	2 (5.7%)	6 (16.7%)	
Grade IV	0	1 (2.8%)	
Radiological outcomes			
AI^a^	26.68 ± 3.65 (19.30–34.10)	27.62 ± 4.59 (19.70–36.50)	0.34
Severin radiological grade^b^			0.26
Grade I	24 (68.6%)	18 (50%)	
Grade II	9 (25.7%)	12 (33.3%)	
Grade III	0	0	
Grade IV	2 (5.7%)	2 (5.6%)	
Grade V	0	1 (2.8%)	
Grade VI	0	3 (8.3%)	
Complications^b^			
Avascular necrosis	0	4 (11.1%)	0.06
Infection	0	1 (2.8%)	0.50
Shenton line disruption	2 (5.7%)	6 (16.7%)	0.13

The bold emphasis indicates statistical significance

*LLD* leg length discrepancy, *AI* acetabular index

*p* value was significant if < 0.05 (Nominal data were compared by chi-squared test while continuous data were compared by Student’s *t*-test)

^a^Data expressed as mean ± SD (range)

^b^Data expressed as frequency (percentage)

**Table 6:** Comparison between patients in group II according to whether a femoral osteotomy was performed or not

	Femoral osteotomy (*n* = 25)	No femoral osteotomy (*n* = 11)	*p* value
Clinical outcomes			
LLD (mm)^a^	3.4 ± 4.72 (0–15)	2.27 ± 4.1 (0–10)	0.56
Modified Severin grade^b^			0.47
Grade I	15 (60%)	5 (45.4%)	
Grade II	6 (24%)	3 (27.3%)	
Grade III	3 (12%)	3 (27.3%)	
Grade IV	1 (4%)	0	
Radiological outcomes			
AI^a^	27.34 ± 4.68 (19.7–36)	28.22 ± 4.52 (21–36)	0.78
Severin radiological grade^b^			0.66
Grade I	13 (52%)	5 (45.4%)	
Grade II	9 (36%)	3 (27.3%)	
Grade III	0	0	
Grade IV	0	2(18.2%)	
Grade V	0	1 (9.1%)	
Grade VI	3 (12%)	0	
Complications^b^			
Avascular necrosis	3 (12%)	1 (9.1%)	0.79
Infection	1 (4%)	0	0.45
Shenton line disruption	3 (12%)	3 (27.3%)	0.34

*LLD* leg length discrepancy, *AI* acetabular index

*p* value was significant if < 0.05

^a^Data expressed as mean ± SD (range)

^b^Data expressed as frequency (percentage)

## Discussion

In the current study, we obtained satisfactory functional and radiological outcomes in most of the patients (regardless of their age) by managing DDH with one stage procedure consisting of OR and DPO, which was performed in all patients; however, in patients above 30 months of age, the need for a concomitant FO was significantly higher. Furthermore, the older patients group needed longer operative time, and their incidence of complications was higher.

Certain anatomical changes of the acetabulum and the femoral head would occur if the femoral head remained dislocated outside the acetabulum; however, these changes could be reversible if the hip was reduced as soon as possible, but the exact upper age at which hip reduction will result in normal acetabular development is uncertain [7, 9]. Many authors suggested that a hip reduced by the age of 4 years could achieve satisfactory acetabular development and growth, and some even extended it up to 8 years of age [14, 25].

Determining the cutoff age to consider a DDH as a late presentation or not is controversial [7, 26]. The debate regarding the optimum age at management could be attributed to the potency of the acetabulum to grow and remodel; the lower limit for acetabular remodeling is 18 months, whereas the upper limit is up to 11 years of age [27, 28]. In the current study, for a single-stage procedure, we included patients below the age of 8 years; they were further divided into two groups, above and below 30 months of age, as was performed in the study by Ning et al. [12].

Correcting the acetabular anatomical characteristics is considered the primary goal of management options, either stimulating the normal acetabulum development by closed reduction of the femoral head or by surgical pelvic osteotomies aiming to improve the acetabulum coverage [29, 30].

Closed reduction in considerably older children with or without adductor tenotomy was reported to offer acceptable results, as long as there is a close follow-up [8]; however, some studies showed that the majority of patients who had successful closed reduction after the age of 18 months required an additional open procedure [31, 32]. This is why most surgeons consider OR for children older than 12–18 months or who failed to achieve a concentric hip reduction by closed maneuvers [9].

Open reduction alone or combined with other procedures such as PO or FO varied in previous studies; in a study by Castañeda et al. [6], including 712 hips with DDH in patients with a mean age of 2.1 years (1–6.5 years), the authors performed OR only in 91 (12.8%) hips—621 (87.2%) hips needed a concomitant PO and a shortening FO was performed in 221 (31%) hips (where all patients were above four years of age). In a study by Charki et al. [33], including 414 hips with DDH, patients had a mean age of 34.6 months (14–96 months), and the type of interventions were as follows: OR only in 18%, OR + FO in 32%, OR + PO in 8%, and the majority had OR + PO + FO (42%); they recommended the addition of a FO in cases with high dislocation (Tönnis 3 or 4) and in cases over 18 months old, while a PO (regardless of sits type) should be added for children older than 36 months and if the AI was > 25, even if the age was as less as 18 months.

On the other hand, if the open reduction was determined, some surgeons advised a whole job single stage surgical procedure, consisting of OR (and capsulorrhaphy), PO, and a FO (if needed) [5, 9, 12]. A single-stage procedure avoids repeated staged surgical intervention with an acceptable outcome. In the current study, all patients underwent OR + DPO, but FO was needed only for patients in group II.

Various pelvic osteotomies were described to obtain previously mentioned goals, the selection of which osteotomy depends on reduction concentricity, patient age, and the status of triradiate cartilage [7, 11]. In the current series, we used DPO exclusively in all patients, which was the same osteotomy performed in 52 hips in the study by Czubak et al. in patients having a mean age of 3.9 years (1.2 to 12.8) [5]. The DPO osteotomy combines the advantages of Salter osteotomy (reorientation of the acetabulum) and Pemberton osteotomy (reshaping of the acetabulum) [34]; furthermore, either anterior or lateral coverage can be obtained by adjusting the osteotomy inner cortical cut [35, 36]. No graft fixation at the osteotomy site was needed owing to the inherent osteotomy site recoil produced by the intact sciatic notch and the posteromedial cortical hinge, and no complications related to fixation devices or the need to be removed [5, 37].

Femoral osteotomy (shortening, derotation, or both) is usually needed if excessive force is required to reduce the hip joint [32]. In the current study, we needed a concomitant FO in 69.4% of patients in group II; however, this was not performed in any patients in group I. Czubak et al. reported performing FO in all hips. However, in a study by Ning et al. [12], who performed a single-stage surgical intervention (using various types of PO) on 864 patients with DDH > 18 months of age(mean age of 5.8 years), they reported performing shortening FO in all hips; however, a derotation was added in selected patients according to the hip anteversion degree measured in preoperative computed tomography (CT) scan. To evaluate factors predicting the need for FO, Sankar et al. [38] evaluated 72 hips with DDH in patients having a mean age of 35.6; they concluded that the patients over the age of 36 months and patients with vertical displacement greater than 30% of the width of the pelvis were more likely to require shortening FO.

Regarding the functional outcomes after single-stage procedures, Ning et al. [12] divided their patients into three groups according to age at surgery: group I: less than 2.5 years; group ii: 2.5–8 years; and group III: > 8 years. After a mean follow-up of 6.2 years (3.2 to 8.9), they reported good or excellent (satisfactory) functional outcomes in a total of 79.4% of patients, with poorer results in group III, and no difference was noted between patients in groups I and II. The same previous observation was concluded from our study, where satisfactory functional outcomes were reported in 94.3% and 80.6% in group I and group II, respectively (all patients were under eight years), with no difference between both groups. Furthermore, after a mean follow up of 4 years (3–9 years), Czubak et al. reported Severin grade I or II (satisfactory) functional outcome in 78.8% of their patients, and no difference was found in patients aged below (group A) or above (group B) 3 years [5]. A systematic review by Wozniak et al. [10] included 23 studies on the outcomes after DPO in DDH. The functional outcomes were reported on 512 hips and were graded as good or very good in 84.8% of the hips by the last follow-up.

Ning et al. reported good or excellent radiological outcomes per Severin classification in 84.7% of their patients [12]. In the current series, most patients reported satisfactory radiographic outcomes according to Severin radiological grades (94.3% in group I versus 83.3% in group II), with no difference between groups. Furthermore, we obtained improvement in the AI measured at the final follow-up in all patients included in the current study compared with the preoperative values (from a mean of 37° to 27.2°, *p* < 0.05) with no difference between both groups. This was consistent with results from the study by Czubak et al., where the AI was improved in all patients compared with the preoperative values (from a mean of 38.8° to 19.5° for group A and from 39.6° to 21.3° for group B), and there was no difference between the AI measured at the last follow-up between both groups. The improvement of AI was reported in 19 studies (636 hips) in the systemic review by Wozniak et al. [10]; it was reduced to ≤ 20° as reported by 16 studies, and the difference between the mean pre- and postoperative AI was 22.5°. Furthermore, the radiographic evaluation according to Severin classification was grade I and II in 81.7% of 410 hips. At a mean follow-up of 9.3 years (6–4 years), Castañeda et al. [6] reported good radiographic outcomes classified as grade I or II per Severin criteria in 80% of the patients; however, they reported that better radiographic outcomes were obtained in patients who underwent open reduction and pelvic osteotomies.

The effect of performing FO on the outcomes was evaluated in a study by Castañeda et al., where the authors evaluated 645 patients with late presenting DDH; 328 hips received a FO compared with 317 hips that did not. The authors reported slightly better functional outcomes in hips which did not have a FO (measured according to Iowa Hip Score); however, they reported no difference in Severin radiographic scores or the incidence of AVN; furthermore, they concluded that FO is not necessarily related to better outcomes [39]. In the current study, we reported that same finding, as there were no significant differences in the functional, radiological, and complications incidence outcomes between patients within group II who had FO and those who did not.

Avascular necrosis (AVN) of the femoral head is one of the devastating complications that could occur after DDH surgical procedures, which varied among studies with an incidence reaching up to 48% [6, 12, 40]. In the current study, we reported an AVN incidence of 5.6%, which occurred exclusively in group II. A similar incidence (5.8%) was reported in the study by Czubak et al. [5], while Ning et al. reported an incidence of 27.4% and reported them as poor outcomes according to the Kalamchi and MacEwen classification [12]. Wozniak et al. [10] reported an AVN incidence of 18.9% from 19 studies (856 hips). The role of PO or FO on the incidence of AVN was controversial; in a systematic review by Kothari et al. [40], evaluating studies on DDH management after walking age, they concluded that the best results (functional, radiological, and lowest AVN incidence) were demonstrated when OR was combined with a PO; they found no evidence to support improvement in outcomes after adding a FO. Charki et al. [33] reported that the radiological results were better in patients where a PO was added; however, contrary to the previous systematic review, they reported that adding a FO contributes to obtaining better functional outcomes and decreasing the incidence of AVN. Furthermore, some technical considerations were suggested to avoid AVN development, including adequate adductor tenotomy, iliopsoas recession, extensive medial capsular release, and division of the transverse acetabular ligament [41, 42].

The current study has some limitations. First, this was a nonrandomized cohort study with relatively few patients. Second, the wide age range of patients included in group II could affect the outcomes. Third, we did not conduct a correlation analysis to determine factors affecting the incidence of complications, especially AVN. Last, a longer follow-up is needed to demonstrate the effectiveness of the surgical technique utilized in the current study.

## Conclusion

One-stage procedure entailing open reduction, Dega pelvic osteotomy, and femoral osteotomy when needed for managing DDH in patients younger than 8 years old revealed acceptable clinical and radiological outcomes. However, the need for a concomitant femoral osteotomy was higher in patients older than 2.5 years, as well as the incidence of complications.

## Data Availability

All the data related to the study are mentioned in the manuscript; however, the raw data are available from the corresponding author and will be provided on a written request.

## Abbreviations

DDH: Developmental dysplasia of the hip
OR: Open reduction
PO: Pelvic osteotomy
FO: Femoral osteotomy
DPO: Dega pelvic osteotomy
LLD: Leg length discrepancy
AP: Anteroposterior
AI: Acetabular index
AVN: Avascular necrosis
